# P73 Cracks in the shield: Comparative evaluation of broth microdilution and Vitek^®^2 for colistin susceptibility testing in a high-AMR tertiary care setting

**DOI:** 10.1093/jacamr/dlaf230.080

**Published:** 2025-12-04

**Authors:** M Nizam Ahmed, Bharat Chandra Das, Parul Singh, Madhavi Kirti, Vanlal Tluanpuii, Ashish Bindra, Subodh Kumar, Sushma Sagar, Kapil Dev Soni, Richa Aggarwal, Keshav Goyal, Gyanendra Pal Singh, Navdeep Sokhal, Samarth Mittal, Kamran Farooque, Purva Mathur

**Affiliations:** All India Institute of Medical Sciences, New Delhi, India; All India Institute of Medical Sciences, New Delhi, India; All India Institute of Medical Sciences, New Delhi, India; All India Institute of Medical Sciences, New Delhi, India; All India Institute of Medical Sciences, New Delhi, India; Trauma Centre, All India Institute of Medical Sciences, New Delhi, India; All India Institute of Medical Sciences, New Delhi, India; All India Institute of Medical Sciences, New Delhi, India; Trauma Centre, All India Institute of Medical Sciences, New Delhi, India; Trauma Centre, All India Institute of Medical Sciences, New Delhi, India; Trauma Centre, All India Institute of Medical Sciences, New Delhi, India; Trauma Centre, All India Institute of Medical Sciences, New Delhi, India; Trauma Centre, All India Institute of Medical Sciences, New Delhi, India; Trauma Centre, All India Institute of Medical Sciences, New Delhi, India; Trauma Centre, All India Institute of Medical Sciences, New Delhi, India; All India Institute of Medical Sciences, New Delhi, India

## Abstract

**Objectives:**

To compare the performance of broth microdilution (BMD), the gold standard method, with the automated Vitek^®^2 system for colistin susceptibility testing. The analysis focused on genus-specific categorical agreement (CA), very major errors (VME), major errors (ME), MIC distributions (MIC50/MIC90) and evidence of heteroresistance among MDR and XDR Gram-negative isolates.

**Methods:**

This observational study was conducted from January to December 2024 at a tertiary care hospital. A total of 2717 non-duplicate Gram-negative isolates were included, comprising *Acinetobacter baumannii*, *Klebsiella pneumoniae*, *Escherichia coli*, *Pseudomonas aeruginosa*, *Enterobacter cloacae* and other less frequently encountered species. Colistin susceptibility was determined using BMD according to CLSI guidelines and Vitek^®^2 with AST-N281 cards. Comparative analysis assessed CA, VME, ME and correlation of MICs using Spearman’s rank. MIC50/MIC90 values were determined, and heteroresistance was inferred from discrepancies in MIC results between the two methods.

**Results:**

By BMD, 93.19% of isolates were classified as intermediate (≤2 μg/mL) and 6.88% as resistant (≥4 μg/mL). Vitek^®^2 reported 95.25% intermediate and 4.75% resistant. Categorical agreement between the two methods varied by genus, ranging from 85.6% for *Enterobacter cloacae* to 100% for rare species. Very major error rates were unacceptably high in several clinically significant genera, including *Enterobacter cloacae* (51.1%), *Escherichia coli* (75%) and *Acinetobacter baumannii* (43.3%), while major error rates were consistently low (0–1.03%). MIC50 and MIC90 values showed species-specific variation, reflecting intrinsic differences in susceptibility patterns. Discrepancies between BMD and Vitek^®^2 suggested heteroresistance, particularly among *Enterobacter cloacae* and *A. baumannii*. Correlation of MICs between methods was poor, with Spearman’s coefficients ranging from 0.02 for *E. cloacae* to 0.48 for *E. coli*.

**Conclusions:**

While Vitek^®^2 demonstrated overall high categorical agreement with BMD, its reliability was undermined by elevated very major error rates, particularly in *Enterobacter cloacae*, *Escherichia coli* and *Acinetobacter baumannii*. These errors risk misclassifying resistant isolates as susceptible, with potential adverse consequences for patient management. Given the critical importance of colistin in treating MDR/XDR Gram-negative infections, BMD confirmation is strongly recommended for invasive or high-risk cases. Furthermore, evidence of heteroresistance underscores the biological complexity of colistin susceptibility and the limitations of automated testing platforms. In high-AMR settings, reliance on automated methods alone may compromise clinical outcomes, reinforcing the need for stewardship-driven approaches incorporating reference-standard testing.

